# Anorexia Nervosa, Body Image Perception and Virtual Reality Therapeutic Applications: State of the Art and Operational Proposal

**DOI:** 10.3390/ijerph19052533

**Published:** 2022-02-22

**Authors:** Massimo Magrini, Olivia Curzio, Marco Tampucci, Gabriele Donzelli, Liliana Cori, Maria Cristina Imiotti, Sandra Maestro, Davide Moroni

**Affiliations:** 1 Institute of Information Science and Technologies “Alessandro Faedo”, National Research Council, Via Moruzzi 1, 56124 Pisa, Italy; massimo.magrini@isti.cnr.it (M.M.); marco.tampucci@isti.cnr.it (M.T.); davide.moroni@cnr.it (D.M.); 2 Unit of Environmental Epidemiology, Institute of Clinical Physiology, National Research Council, Via Moruzzi 1, 56124 Pisa, Italy; liliana.cori@ifc.cnr.it (L.C.); cristina.imiotti@ifc.cnr.it (M.C.I.); 3 Department of Health Science, University of Florence, 50134 Florence, Italy; gabriele.donzelli@unifi.it; 4 Department of Developmental Neuroscience, IRCCS Stella Maris Foundation, 56128 Calambrone, Italy; sandra.maestro@fsm.unipi.it

**Keywords:** anorexia nervosa, body image, virtual reality, systematic review

## Abstract

Anorexia Nervosa (AN) patients exhibit distorted body representation. The purpose of this study was to explore studies that analyze virtual reality (VR) applications, related to body image issues, to propose a new tool in this field. We conducted a systematic review in accordance with the Preferred Reporting Items for Systematic Reviews and Meta-Analyses (PRISMA) guidelines. PubMed, EMBASE, Scopus, and Web of Science databases were explored; the review included 25 studies. Research has increased over the last five years. The selected studies, clinical observational studies (*n* = 16), mostly concerning patients’ population with AN (*n* = 14) or eating disorders (EDs) diagnosis, presented multiple designs, populations involved, and procedures. Some of these studies included healthy control groups (*n* = 7). Studies on community sample populations were also selected if oriented toward clinical applications (*n* = 9). The VR technologies in the examined period (about 20 years) have evolved significantly, going from very complex and bulky systems, requiring very powerful computers, to agile systems. The advent of low-cost VR devices has given a big boost to research works. Moreover, the operational proposal that emerges from this work supports the use of biofeedback techniques aimed at evaluating the results of therapeutic interventions in the treatment of adolescent patients diagnosed with AN.

## 1. Introduction

Anorexia Nervosa (AN) patients exhibit distorted body representation related to perceptual and cognitive-emotional issues. Experimental paradigms are needed to manipulate the spatial content of these representations. Virtual Reality (VR) technology that implements full-body experiences may prove useful in clinical application [1].

AN is a condition that mainly impacts adolescents and can be associated with functional impairment. AN is more frequent in females and contributes to psychological, and biological dysfunctions [2]. The lifetime prevalence of AN in adults is about 0.6% (0.9% in females and 0.3% in males) [3]. Neuropsychological investigations have found that AN patients are impaired in different cognitive domains, such as visuospatial abilities [4,5], empathic abilities [6,7], executive functioning [8], and central coherence [9].

Among executive functioning, a specific weakness in set-shifting or cognitive flexibility has been consistently reported in AN patients [10]. Impaired behavioural response shifting has been related to abnormalities in the fronto-striato-thalamic circuitry [11]. Whereas several studies have provided evidence that cognitive alterations are present during starvation, empirical support that documents these deficits outside the AN acute phase of malnutrition is more elusive. Reduced set-shifting and weak central coherence are thought to be part of the eating disorder endophenotype [9]. It has been proposed that these neuropsychological dysfunctions may have specific links with the core clinical characteristics of AN. In particular, impaired set-shifting may be linked with the cognitive and behavioural pattern of inflexibility [10]; weak central coherence may be linked with the excessive preoccupation with detail of body parts and weight [12], visuospatial deficits may be related to the distortion of body image (BI) [13] It has been shown that restrictive (AN-R) or purging subtypes of AN may have different cognitive profiles, with the first characterized by reflective cognitive style and the latter by impulsivity [13,14]. Previous studies have documented in particular an inaccuracy in the estimation of one’s own body parts in patients with AN, suggesting that disordered body perception may be a central aspect of AN. Disordered body perception is one of the principal risk factors [15], one of the main symptoms in AN (linked to depression and anxiety), a significant maintenance and prognosis factor, a predictor of relapse. In sum, many studies have confirmed the strong relationship between AN and BI; when dealing with patients with AN, trying to improve their BI is a commonly used intervention approach.

Ziser and colleagues (2018) carried out a systematic review, conducted according to the PRISMA statement, about the evidence on BI directed interventions in AN. Targeting BI disturbances may be efficacious [16], and exposure therapy is a potential method for the treatment of AN and eating disorders (EDs): VR exposure could improve accessibility and feasibility of exposures in the clinical setting [17].

In this context, Clus and colleagues (2018) [1] conducted a broad analysis of studies on the use of VR in patients with various EDs (publication date of the articles included range from the year 1998 to the first period of 2016), showing that VR is an acceptable and promising therapeutic tool for patients with EDs. Commercialized VR technology is increasingly accessible to the general public and medical research, especially in mental health, with diagnostic, therapeutic, and preventive aims. The use of VR in the evaluation or treatment of patients with EDs is being led by European teams, and most of the technologies used to enable immersion in the virtual environment have been developed in the United States. The heterogeneity of the populations studied, the studies’ objectives and the content of the VR protocols make interpretation of the results difficult. Moreover, many studies concluded without differentiating the subtypes of EDs [1] even if knowledge of both transdiagnostic and specific mechanisms in ED subtypes is of relevance for clinical practice [18].

Autonomic imbalance and its connection with interoception has an important role in AN symptomatology [19]; this altered sense of the physical body in AN contributes generating very specific emotions and behaviours [20]. A previous study suggested that two main neural networks—the limbic and the frontal—could be particularly relevant in AN [21].

A recent review by Riva and colleagues (2021) outlined that VR could be used to modify the allocentric memory of the body, to improve the processes of multisensory integration through multisensory body illusions and to reduce attentional biases to body-related stimuli [22]. The hypothesis developed to explain the perturbations of BI in patients with EDs is the allocentric lock hypothesis [23]. The use of VR could make it possible to unblock this transmission. The theory of objectification as a specific cognitive process is cited to understand these perturbations: a person internalizes an objectified self-image when using a reference allocentric frame (observer mode) to recall the events in which they evaluate themselves based on body appearance. The validity of using a virtual environment in a population can be judged by the individual’s emotional reactions. Unique exposure to virtual silhouettes of the patient or mannequin increases anxiety and changes mood, reproducing physiological reactions to a real situation. The repetition of VR sessions with modules of exposure to silhouettes of the patient or mannequin reduces negative emotions, by progressive attenuation of the anxiogenic response [1]. As explained by Dakanalis and colleagues “allocentric lock” model of EDs and AN in particular [24] provides a rich conceptual framework for understanding the source of BI disturbance. People are generally unable to accurately determine their own body measurements and translate this knowledge to identify a model/avatar that best represents their own body. This inability has been related mainly to health problems as in the first place AN [25].

The “rubber hand illusion” (RHI) was the forerunner of current VR research: Since Botvinick and Cohen’s original publication [26] revealing that observing a rubber hand being stroked or touched synchronously with one’s own hidden hand generates the illusion in people that a rubber hand is part of their body (RHI), there has been increasing research interest in the study and modulation of the brain’s body representation. More recently, an increasing body of pioneering research has endeavoured to adapt the RHI to the entire body (body-swap illusion) using the same principles (as visuo-tactile synchrony between the real body and the seen surrogate body) [27]. This research revealed that the embodiment in a virtual body substituting ones’ own body in VR with visuo-tactile stimulation alters body percept [28]. A recent study [29] has shown that the body-swap illusion was able to induce an update of the negative stored representation of the body. Although true that the studies on body-swap illusion can be classified in terms of the main cross-modal stimuli provided [30], in the EDs field, all the studies conducted are based on visuo-tactile triggers for body-swap. In fact, visuotactile and proprioceptive integration are critical in perceiving our body highlighting the issue of the multisensory and affective impairment of body perception and representation [30,31,32].

In a few studies, researchers have considered the inclusion of bio-feedback in their therapeutic application of VR to generalize anxiety [33] and EDs [34]. Indeed, physiological parameters might be computed in real-time on the study participants, e.g., through wearable or contactless sensors, and used to control the content and appearance of the proposed virtual experience. In most cases, skin conductivity, a parameter related to arousal, has been used to control and deliver virtual images related to the arousal status. In the studies, the ultimate goal is to train the participants to regulate their physiological parameters using the VR experience as a gauge. In this way, the main obtained effect is relaxation and stress reduction, which has proven to be effective as a complementary approach to EDs treatment [35]. Moreover, given recent perspectives [36], however, the use of bio-feedback for acting and transforming the body image seems not to have been accurately investigated. Thus, further research in this area, including studies attempting to produce changes in perceptual and affective body-image components specifically in anorexia AN are required. The improvement of VR therapeutic applications is desirable.

The present research can be subdivided into the following main domains and objectives:

− To review the literature on BI disturbances and VR clinical applications related to patients with AN;
− To propose a new tool and a new application of VR, AN population therapy oriented, related to BI overestimation and dissatisfaction.

In particular, our proposal aims at making innovative use of bio-feedback, based on galvanic skin response (GSR) real-time analysis to provide a controlled VR experience able to drive a beneficial change in the body image.

## 2. Materials and Methods

For the present systematic review, the Preferred Reporting Items for Systematic Reviews and Meta-Analyses (PRISMA) statement was adopted [37].

### 2.1. Search Strategy

This review was performed by searching four different electronic databases, PubMed, Embase, Scopus and Web of Science. Anorexia nervosa, body image, VR were the keywords in the following search query: “(Anorexia Nervosa) AND (Body Image) AND (VR)”. Studies written in English were included and reviewed; letters to the editor and abstracts were excluded. The search of the four databases was conducted without any time limitations on 30 December 2020 by G.D.

### 2.2. Criteria for Eligibility

The following inclusion criteria were adopted: AN or other EDs considering AN; clinical population or not clinical population but the studies considered had to be oriented towards the implementation of a tool for BI disturbances in AN treatment; BI and VR should be specifically mentioned; BI and VR application results can be evaluated via quantitative or qualitative methods.

### 2.3. Study Selection

**Figure 1:**
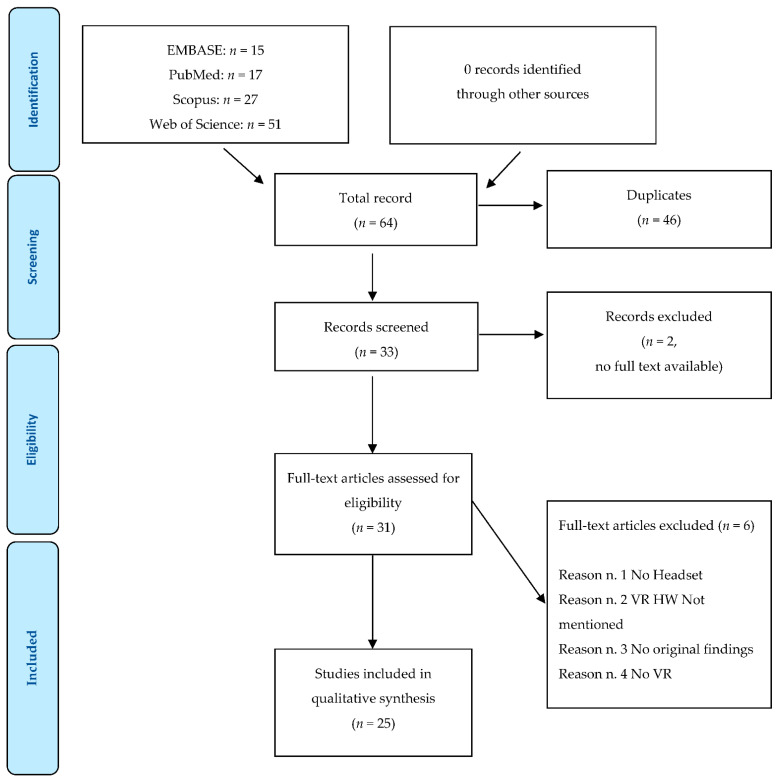
PRISMA flow diagram.

After removing duplicates, three researchers, who are among the authors of the paper (O.C., M.M., and M.T.), independently evaluated titles and abstracts based on the eligibility criteria. The articles selected by the three reviewers were employed in the next phase, and the full text was read. All the authors thoroughly read the articles specified in the first phase in equal proportion, deciding to accept or reject the papers. In case of conflicts, the three authors discussed together and, if the agreement was not reached, a fourth author (S.M.) expressed the final judgment. The selection process is shown in Figure 1, which utilizes the flow chart provided by the PRISMA guidelines. We used the Newcastle-Ottawa Scale (NOS) to assess the quality of each study (http://www.ohri.ca/programs/clinical_epidemiology/oxford.asp, accessed on 15 September 2021). A score of a maximum of 9 stars was assigned to each study and reported in Table 1, where the characteristics of the studies are described. For case report studies, we used a dedicated scale published by Murad et al., 2017 which assigns a maximum of 8 points for each study [38].

### 2.4. Data Extraction

Relevant features were extracted and, specifically, the following information was considered: the methodology; the characteristics of the participants involved in the study, whether specific devices were tested; which tools were used. These relevant data were included in table form (Table 1) to obtain a synthetic framework of all articles read in full by authors. This table format enabled the authors to complete a cursory overview of the materials selected in the first phase.

### 2.5. Virtual Reality Conceptual Dimensions and Applications to Body Image

To be able to analyse the VR application, the main methodological issue is the extent of the concept. The VR instruments linked to BI measurement are intended to gather information about understanding and knowledge of the structure of the tools and the experimentation.

## 3. Results

### 3.1. Search Results and Study Characteristics

The flow diagram in Figure 1 describes the article selection process we followed for incorporating the studies in the present review. Searching the four databases mentioned in the Methods 110 articles were identified. From these first records, we removed 46 duplicates, leaving 64 for further review. The number of included studies was reduced to 33 after screening the titles and abstracts and applying the following exclusion criteria:

- Generic studies
- Editorials
- Studies without original findings
- Studies where there were no VR tools

The remaining articles underwent a full-text evaluation, bringing the total number down to 25 published articles. By the end of the identification process, we had removed more than half of the reports from the amount we initially identified. Table 1 summarizes the main characteristics of the studies and the VR tools included in this review in reverse order of publication.

### 3.2. Geographical and Timeline Distribution

**Figure 2:**
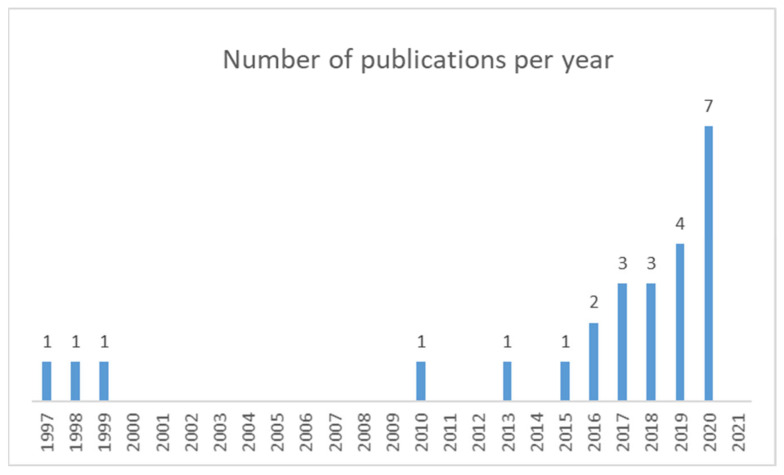
Timeline distribution of the articles included in the systematic review.

Looking at the timeline distribution of the articles, Figure 2 shows that research has increased over the last five years, during which time over 75% of the studies were published.

**Figure 3:**
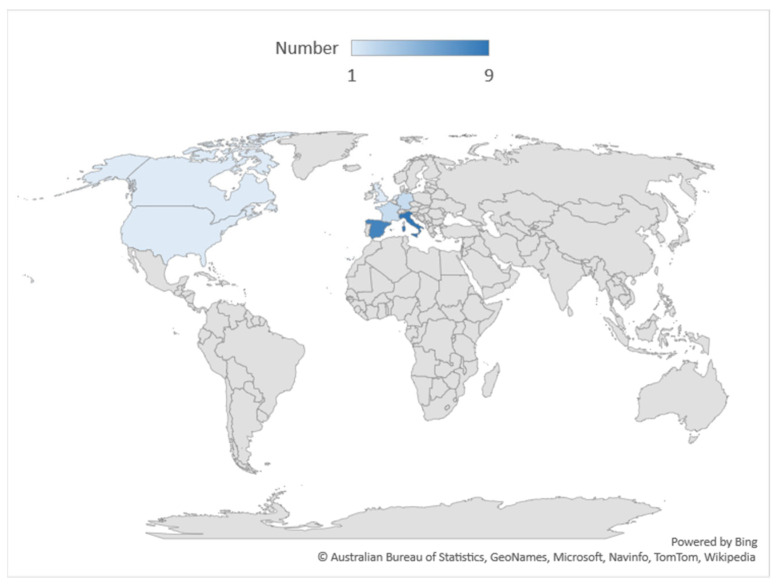
Geographical distribution of the studies included in the systematic review.

The articles included in this systematic review concern studies carried out in Italy (*n* = 9), Spain (*n* = 8), France (*n* = 2), Germany (*n* = 2), the UK (*n* = 1), the USA (*n* = 2), and the Netherlands (*n* = 1). Figure 3 shows the geographical distribution of surveyed countries.

### 3.3. Study Design and Population

The 25 studies selected presented multiple designs, populations involved, and procedures. Many selected studies were clinical observational studies (*n* = 16), primarily concerning patients population with anorexia nervosa diagnoses (*n* = 14) or EDs diagnoses [39,40]. Some of these clinical studies included healthy control groups (*n* = 7) [32,39,41,42,43,44,45]. Single clinical cases (*n* = 6) [46,47,48,49,50] were also included in the present review. In the present review, the authors also selected studies on community sample population because this kind of research was in any case-oriented toward the possible clinical application in the field of AN and EDs treatment (overall *n* = 9). The general population studies were nine in total, but some of them included volunteers with high body concerns [51,52].

### 3.4. Avatar and Multisensory Integration: Stimulus Generation, Technical and Emotional Setup

Irvine et al. (2020) [51] tested the efficacy of a training program delivered in VR to modify BI in female volunteers with high BI concerns. In a 4-day training programme in VR, participants categorized a series of 3D models (thin or fat). One group was presented with the stimuli briefly, while the other intervention group had no time limits, and inflationary feedback to shift their categorizations of the stimulus models towards higher body mass indexes (BMIs) was given. The “reference frame-shifting approach”, which is focused on the reorganization of body-related memories [23] involves the VR adaption of the imagery rescripting method aimed at changing the meaning linked with negative memories of the body. Riva et al. (2018) [36] developed a specific BI rescripting protocol: a sensory training to ‘unlock’ the body memory by increasing the contribution of new somatosensory information related to the negative memory [36]. Riva and his team pioneered the use of avatars to measure and treat body representation disturbances [50]: patients were asked to select among nine avatars ranging from underweight to overweight, indicating how they perceive themselves [49]. After the discussion of their emotions emerged in the first phases (exposition to digitalized photographs of their real bodies in different formats) with clinicians, patients modelled their perceived BI using an avatar and compared it with their actual and ideal BI.

In the Neiret et al. study [53], a virtual representation of participants’ internal image of their body shape was offered. A virtual body corresponding to the representation they had of their ideal body was also created, and another virtual body based on their real body measures was built. Furthermore, in this case, participants saw three different virtual bodies from an embodied first-person perspective and a third-person perspective. Participant bodies were scanned and generated as an avatar. Two alternatives were generated for each body, increasing or decreasing its size. Afterwards, participants had to choose which is their body between the three proposed. In the study of Provenzano et al. [41], combined virtual reality and multisensory bodily illusion were used to characterize and reduce the perceptual (body overestimation) and the cognitive-emotional (body dissatisfaction) components of BI distortion in AN. Interpersonal multisensory stimulation (IMS) was applied to the avatar, reproducing the participant’s perceived body. The two avatars reproduced increases and losses of 15%, all presented with a first-person perspective (1PP). Participants had to choose their avatar respecting their actual body size. After that, they had to complete a set of tasks and experience a set of both synchronous and asynchronous stimuli with three different body size avatars.

New technological tools such as virtual reality (VR) applications have improved the feeling to be the avatar with the immersive conditions. Head-tracking technology allowed for the implicit measurement of explicit choices of patients. The retrospective study by Fisher et al. [54] examines the hypothesis that VR with standardized 3D avatars would improve BI perception and then BI evaluation by adolescents with AN, compared to the paper-based figure rating scales (FRS). The creation of personalized avatars to simulate realistic changes in body size is useful when studying self-perception of body size. Hudson and colleagues [55] explored this topic in young adult women, using a generalized line drawing scale and several types of personalized avatars, including 3D textured images presented in immersive virtual reality (VR). Each participant views both 2D and 3D avatars of their body along with the other three avatars with different body sizes. The order of the avatar showing is random. Body perception ratings using generalized line drawings were often higher than responses using individualized visualization methods.

Mölbert et al. [42], in a case-control study aimed at disentangling the components of BID in AN, investigated 24 women with AN and *n* = 24 controls. Using different psychophysical tasks, participants considered their actual and their desired body shape testing for general perceptual biases. Based on a three-dimensional (3D) body scan, the researchers offered virtual 3D bodies in a virtual reality mirror scenario. The experiment is composed of three parts: (a) body scanning; (b) moving inside a virtual scene and observing the avatar in a mirror; (c) observing the 2D version of the avatar on Desktop. In Rubo et al. [43] the avatar is calibrated with the body size of the participant and is then slightly incremented. After the calibration phase, participants observe themselves through a mirror and have to fulfil simple tasks such as touching their hips and stomach and walking around a table.

Fonseca Baeza et al. [56] presented the study protocol of a novel Virtual Reality (VR) multisensorial paradigm to assess and treat BID. A female standard virtual body has been developed for all participants to see it in a first-person point-of-view or a third person point-of-view in a mirror. The participant will not be able to see the face or the hair of the avatar. It will be possible to choose the body size along a continuum from 133 cm of waist and 151 cm of hips (extremely overweight) to 65 cm of waist and 88 cm of hips (thin), covering a body BMIs rate from 42.5 to 12.5. The avatar was presented with a body mass index (BMI) similar to the participant’s one. Afterwards, it is increased and decreased by 2-point BMI, and the participant is asked to modify the avatar until they consider that the avatar coincides with their actual abdomen. The control group condition task consisted of making slow movements observing the virtual abdomen. This protocol allows the development of a more realistic corporal representation. In Corno et al. [57], a sample of 27 community women recreated in VR their perceived body in both an allocentric and egocentric perspective. Attitudinal indexes of BID were assessed through validated questionnaires. The third-person view (TPV) is obtained by a mirror located in the virtual scene. Starting from a body with a BMI of 20.5, participants have to indicate how to modify the avatar to recreate a body size corresponding to the one perceived both in first-person view (FPV) and TPV. Virtual bodies (presented in a continuum from extreme underweight to morbid obesity) were viewed without their heads and dressed in blue shorts and black crop top.

To modify body-size perception through an illusion of ownership over a virtual body, Buche et al. [58] proposed to couple a tactile stimulation when viewing an avatar from a third-person perspective (a condition known to produce this kind of illusion). This application offers the possibility to choose between avatars of different builds and to perform morphing to reduce the avatar’s body. Moreover, the application allows to implicitly measure how people perceive their body size from an affordance estimation task in which people have to appreciate if they can pass through doors of different sizes without twisting their shoulders. Buche et al. [58] carried on a 3D virtual environment for inducing body ownership illusion implementing an experiment on 16 female participants. They performed the affordance estimation task five times: the first time before being exposed to their chosen avatar to get a baseline measure, and the other four times after exposure to their avatar in different situations. These different situations are defined by the crossing of two experimental factors: morphing (presence or absence) and simultaneous visuotactile stimulation (presence or absence). In Ferrer Garcia [59], college students (5 males) were exposed to an immersive VR environment, where the illusion of ownership of a virtual body induced using visuomotor synchronization assessed the ability of a VR-based software to produce body anxiety responses in a non-clinical sample. BMI, drive for thinness and body dissatisfaction were assessed before exposure, while body anxiety, fear of gaining weight and ownership illusion were assessed after exposure to each avatar. In Gutiettez-Maldonado and colleagues [48], a 22-year old female anorectic patient underwent a VR and Experiential Cognitive Therapy (ECT) to address both body experience disturbances and motivation for change. Exposure to an embodied avatar has been used by Porras-Garcia et al. [46]. The procedure consisted of five sessions in which a patient suffering from AN embodied an avatar of progressively increasing BMI. Porras-Garcia et al. [52] used a VR-based embodiment procedure in which participants owned an avatar with their own body measurements, in comparison to a larger-size one [52].

### 3.5. Intervention Evaluation Studies

In most of the analyzed studies, intervention groups experienced reductions in BI concern and, in the groups with longer stimulus presentation times, these reductions were consistent with a clinically meaningful effect. Third-person perspective allowed them to perceive the real body shape without applying the negative prior beliefs associated with the self; this resulted in a more positive evaluation of their body shape. Only in Fisher et al. [54] and in Hudson et al. [55], are results of BID evaluation by VR standardized 3D avatars comparable to those obtained by paper-based FRS, and presentation in immersive VR may not be essential.

Full body ownership illusions in VR can be robustly induced by providing congruent visual stimulation, and that congruent tactile experiences provide a dispensable extension to an already established phenomenon. In Rubo et al. [43] visuotactile congruency indeed does not add to already high measures for body ownership on explicit measures but does modulate movement behaviour when walking in the laboratory. Participants who took ownership over a more corpulent virtual body with intact visuotactile congruency increased safety distances towards the laboratory’s walls compared to participants who experienced the same illusion with deteriorated visuotactile congruency. This effect is in line with the body schema more readily adapting to a more corpulent body after receiving congruent tactile information. The researchers concluded that the action-oriented, unconscious body schema relies more heavily on tactile information compared to more explicit aspects of body ownership. In Corno et al. [57], in line with the allocentric lock hypothesis, the results confirmed the existence of two different mechanisms underlying BID: the egocentric and the allocentric frame. In Buche et al. [58], the REVAM application links two main aspects of body size perception: the first one focuses on its modification and the second one concerns its assessment indicated that exposing people to a virtual body reduced in could be a way to modify body size perception, at least temporarily.

In Ferrer Garcia et al. [59], students reported higher levels of body anxiety and fear of gaining weight after owning a 40% larger virtual body, in particular, the students with higher scores in the scales of body dissatisfaction and drive for the thinness of the validated tests used. In the research of Conxa Perpiñá et al. [40], improvement was maintained in post-treatment and at one-year follow-up. The results reveal the advantage of including a treatment component addressing BI disturbances in the protocol for the general treatment of EDs.

Gutiettez-Maldonado and colleagues [48] detailed the characteristics of the ECT, an integrated approach ranging from cognitive-behavioural therapy to virtual reality (VR) sessions. Multisensory bodily illusions since the pivotal work of Botvinick and Cohen [26] have been used to investigate the plasticity of bodily experience and representation [60]. Keizer and colleagues showed in a case-control study that patients with AN experienced a stronger RHI when compared to healthy controls. Moreover, RHI was able to induce a decrease in the overestimation of hand width only in AN subjects [29]. Keizer and colleagues [32] also showed that after the embodiment procedure, participants with AN exhibited a decrease in the overestimation of their bodies and part of the body lasting for two hours. Porras-Garcia et al. [52] carried out several studies using a VR based embodiment method. Results showed a reduction in the body related anxiety, fear of gaining weight, body-related attentional bias and BI disturbances [46].

### 3.6. The VR Technologies

In the examined period (about 20 years), VR technologies have certainly evolved a lot. We have gone from very complex and bulky systems, which required very powerful computers, to cheaper and much more agile systems. The advent of low-cost VR devices such as the Oculus Rift or HTC Vive has undoubtedly given a significant boost to research works that use virtual reality technologies.

Among the works analyzed, 10 use the Oculus Rift (Dk1 and Dk2), five the HTC Vive, five other devices (generally older and more expensive) while the remaining five did not specify the system used.

The display of an avatar (especially in TPV form, present in 12 articles among those selected) controlled by the subject’s posture requires a device capable of detecting and tracking it, such as the Optitrack Motion Capture Systems, reported in Article 2. Surely the Kinect device (created by Microsoft to interact with video games) has greatly facilitated, and economically, this type of task. In the works analyzed, three [55,57,58] use the Kinect for movement tracking. The Kinect can also be used to perform a body scanning similar (albeit with a lower resolution) to that obtainable from dedicated devices, useful where the experiment provides for a pseudo-realistic representation of one’s body [55].

Some tasks of the various experiments require additional hardware. Tasks that involve gaze analysis require an eye tracking device, such as the VR HMD FOVE [46,60,61]. As for systems that provide approaches related to third body ownership illusion, some works use input devices such as Razer Hydra [32,58,60], to track the movement pertaining to tactile stimulation in the 3D setting. Reference [51] on the other hand, envisages a more complex system for tactile stimulation, using a self-built system based on vibration actuators connected to Arduino boards.

Among the various software (SW) tools used, it should be noted that Makehuman [41,47,56,57] is often used for the preparation of avatars. This is an open-source tool for creating 3D characters complete with rigging, which can be easily imported into various game engines. As for the game engines used (among the few articles that report it) for the preparation of 3D environments, the most common is undoubtedly Unity 3D [32,43,46,47,52,55,57,61]. Even with the free version, this engine can build complete and functional systems on many platforms. Furthermore, compared to other game engines (e.g., Unreal) [51] it has a certainly sweeter learning curve.

### 3.7. Technical Details on the Hardware/Software Used

#### 3.7.1. VR Headset

VR Headset allows users to experience the virtual environment immersively. Through them, users have a direct stereoscopic view of the environment that provides a real sense of space and distances and, thanks to the accelerometer sensors equipped on the headsets, head movements are translated into camera movement. Most of the works analyzed in this review, which exploits these features to provide a set of stimulations to the participants, use commercial devices: 10 of them are based on Oculus Rift, five on HTC Vive. Less common (or prototype) devices have been used in some of the works, such as the Thunder 400/C. Finally, some works do not report any details on VR Hardware used.

Oculus Rift is a line of virtual reality headsets developed and manufactured by Oculus VR, a division of Facebook, Inc., released in 2016. Despite Oculus Quest 2 being the only model currently produced, Oculus VR has developed and distributed several different models: DK1 (development kit), DK2, Oculus Rift, Oculus Go, Oculus Rift S, Oculus Quest and Oculus Quest 2. DK1 and DK2 are the only pre-production models, among the five developed, which were shipped to backers; the DK1 in 2013 and DK2 in 2014, intended to provide developers with a platform to develop applications before the final release. The Rift DK1 was released in 2013 and used a 7-inch screen with a 1280 × 800 resolution (640 × 800 effective) per eye. It included interchangeable lenses that aim to allow for simple dioptric correction. The DK2 (2014) featured several key improvements over the first development kit, such as having a higher-resolution (960 × 1080 per eye) low-persistence OLED display, higher refresh rate, positional tracking, a detachable cable, and the omission of the need for the external control box. The first consumer version, the CV1, was released in 2015 and featured per-eye displays with a 1080 × 1200 resolution, running at 90 Hz, 360-degree positional tracking, integrated audio, increased positional tracking volume, and a better focus on ergonomics. The last Rift version was the S: it has a 1280 × 1440 display running at 80 Hz display and a slightly larger field of view than that of the CV1. The Rift S tracks the position of itself and its controllers in 3D space using a system known as Oculus Insight, which uses the five cameras on the HMD to track points in the environment and infrared LEDs on the controllers, information from accelerometers in both the HMD and controllers and computer vision to predict what path the HMD and controllers are most likely to take.

Oculus Go, Oculus Quest and Oculus Quest 2 are the portable versions of Oculus Rift; none of them is used in the works analyzed for this review which use Oculus Rift as a headset. Thus, their specs will not be described.

HTC Vive is a virtual reality headset produced by HTC Corporation and presented during the Mobile World Congress keynote in 2015. HTC Vive has been developed within a collaboration with Valve Corporation, to implement the SteamVR hardware and software ecosystem. The HTC Vive implements “room-scale” virtual reality. In general, VR applications can allow users to walk freely around a predetermined area or, to avoid motion sickness, to constrain them to a set of stationary positions. The controllers and headset use a positional tracking system known as “Lighthouse” based on LED lights, and two infrared lasers on the system’s base stations. The headset is connected to the Windows PC using an adapter called “link box”. Link Box is composed of a USB 3.0, an HDMI and power connectors. In 2018 HTC presented an upgraded Vive model known as HTC Vive Pro, having higher-resolution displays, now at 1440 × 1600 resolution per eye. Vive Pro Eye, released in 2019, added built-in eye tracking. In 2021, HTC released the Vive Pro 2, which upgrades its screens to 2448 × 2448 resolution per eye (marketed as 5 K resolution), with a 120-degree field of view and 120 Hz refresh rate. Other, more recent models are the Vive Focus (now at version 3), which offers has a per-eye resolution of 2448 × 2448 at 90 Hz with a 120-degree field of view, and the stand-alone Vive Cosmos (similar to Oculus Quest), which has a 2880 × 1700 display.

#### 3.7.2. Other Hardware/Software (HW/SW) Devices

Razer Hydra is a motion and orientation controller developed by Sixense Entertainment, in partnership with Razer USA. It uses a weak magnetic field to detect the absolute position and orientation of the controllers with a precision of 1 mm and 1°; it has six degrees of freedom. It was used in some of the works of this review, together with Oculus Rift DK2 (which has no controllers), for implementing the third body illusion.

Kinect is a motion-detection device produced by Microsoft in three versions starting from 2010 (V1, V2, Azure). The devices contain infrared cameras and projectors that map depth through structured light (version V1) or time-of-flight calculation (version V2 or Azure). Kinect can perform real-time gesture recognition, body skeleton detection, and face detection (V2 and Azure). It is connected to the computer via a standard USB 3 connection. The most recent version (Azure) requires the latest generation Nvidia GPU.

MakeHuman is an SW designed for the prototyping of photorealistic humanoids, free and open source. MakeHuman allows designing a great variety of male and female characters, starting from a unique starting base mesh. Each unique character design is obtained through linear interpolation. By defining four morphing targets (baby, teen, young, old), MakeHuman can design all the intermediate ageing stages automatically. Using this SW, it is possible to reproduce a considerable number of different characters. It has been used in four works of this review for avatar’s generation.

## 4. Discussion

Overall, this review provides evidence of the usefulness of virtual body ownership illusions to reduce weight- and body-related anxiety responses and opens the door to its therapeutic use in patients with anorexia nervosa. The present research aimed to review the literature on body image (BI) disturbances and virtual reality applications related to patients with AN feature; to propose a new tool and a new application of Virtual Reality AN population therapy oriented. The chosen search terms, anorexia nervosa, body image, virtual reality with the Boolean operator “and” were used precisely to restrict interest in those research papers that simultaneously considered these three concepts that are important to the authors in an operational manner. However, there could be the possibility of not having included all the studies in the sector of interest. The selection process was shown in Figure 1 which utilized the flow chart provided by the PRISMA guidelines. The objective of Clus and colleagues review (published in 2018; articles included in the review 1997–2016) [1] was to provide a review of the applications of VR in patients with EDs. That review included studies in the area of virtual work on patients’ BI and exposure to virtual food stimuli. On the other hand, the present research focused on the problems inherent BI in patients with AN and in experiments involving healthy people who were oriented towards methods of treatment for AN. Furthermore, among the present research objectives, there was the implementation of a virtual reality tool to be tested in the near future on adolescent patients in care of two clinics of the national health system present on our territory, as explained in the next section.

Research in this specific field has increased over the last five years, during which time over 75% of the studies were published. The analyzed articles concern studies carried out in Italy (*n* = 9) and Spain (*n* = 8). The 25 studies selected presented multiple designs, populations involved, and procedures. Many selected studies were clinical observational studies (*n* = 16). In most of the analyzed studies, intervention groups experienced reductions in BI concern and in the group with longer stimulus presentation times these reductions were consistent with a clinically meaningful effect.

In recent years, the research interest in the multisensory integration processes and the rapid technological development of the VR field has led to the possibility of using VR for correcting a dysfunctional body experience in patients with AN using realistic avatars. Third-person perspective allowed them to perceive the real body shape without applying the negative prior beliefs associated with the self. Only in Fisher et al. (2020) and Hudson and colleagues (2020) results of BID evaluation by VR standardized 3D avatars are comparable to those obtained by paper-based FRS and presentation in immersive VR may not be essential [54,55].

Among the works analyzed, 10 use the Oculus Rift (Dk1 and Dk2), five the HTC Vive, five other devices (generally older and more expensive). The display of an avatar (especially in TPV form, present in 12 articles among those selected) controlled by the subject’s posture requires a device capable of detecting and tracking it, such as the Optitrack Motion Capture Systems. Surely the Kinect device has greatly facilitated, and economically, this type of task [55,57,58]. Some tasks of the various experiments require additional hardware. Tasks that involve gaze analysis require an Eye Tracking device [46,52,61]. As for systems that provide approaches related to third body ownership illusion, some works use input devices such as Razer Hydra [32,58,61], to track the movement related to tactile stimulation in the 3D setting. Among the various SW tools used, it should be noted that Makehuman [41,47,56,57] is often used for the preparation of avatars.

Experiments with a fake body part have revealed how the brain becomes confused during a party trick known as the rubber hand illusion (RHI). Under the illusion, people feel that a rubber hand placed on the table before them is their own, a bizarre but convincing shift in perception that is accompanied by a sense of disowning their real hand. The studies on body-swap illusion can be classified in terms of the main cross-modal stimuli provided [30]. In the EDs field, visuotactile and proprioceptive integration is critical in perceiving our body, highlighting the issue of the multisensory and affective impairment of body perception and representation [30,31,32].

The RHI is the most complete investigated body ownership illusion. Botvinick and Cohen found that participants perceived the rubber hand as part of their own body, attributing the tactile stimulation they felt to the rubber hand. Moreover, Botvinick and Cohen employed a behavioural measure later termed proprioceptive drift, which involved blindly pointing to one’s hand before and after the brushing. A significantly larger displacement of the perceived location of the participant’s real hand toward the rubber hand after synchronous compared to asynchronous stroking was observed. Physiological measures have also been employed to capture the subtle differences in individuals’ experience of body [26]. First employed by Armel and Ramachandran [62], skin conductance responses (SCRs or GSR, galvanic skin response) have become used as an autonomic RHI measure [63]. SCRs are changes in skin conductance resulting from increased sweat gland activity induced by discrete stimuli. These stimuli could be painful movements, knives or hammers that threaten the integrity of an embodied rubber hand and induce SCRs that are like the SCRs resulting from threatening one’s real hand. This measure is an index for arousal as it captures responses occurring at an unconscious level. The RHI has also been induced in VR, where individuals experience illusory ownership over virtual bodies/body parts following synchronous visuotactile or visuomotor feedback. The provision of sensorimotor contingencies is an important factor to both immersion and body ownership. Therefore, body ownership and immersion seem to be interconnected in multiple ways [64].

## 5. Implications/Proposal

The selected papers were oriented for possible use within therapies for AN. Several of them adopt embodiment methods to induce the identification of the subject with the avatar in the virtual scene. Based on the analysis of these systems, and in light of the technical considerations reported above, the authors propose a system that combines the analyzed techniques with biofeedback. Biofeedback (or biological feedback) is a psychophysiological intervention method that can be framed in the field of applied psychophysiology. Psychological learning theory based on operant conditioning is the basis of biofeedback therapy. This is made up of three essential characteristics (positive reinforcement, conditional reinforcement and generalization), which are strictly involved in the learning process. It involves a process of teaching the patient to recognize and subsequently control a physiological function. Biofeedback can be used alone or as part of a general treatment program [65]. Basically, with biofeedback systems the subject learns to recognize, correct and prevent the physiological alterations underlying various pathological conditions, contributing to a positive course of his pathology. Typically, a specific bodily function such as muscle tension or some parameters related to electroencephalographic signals is monitored with the use of special sensors. The detected signals are then appropriately mapped into visual or auditory stimuli. The patient, through a training process, at least initially guided by an operator, can thus adopt control strategies to learn to control the monitored function voluntarily. By controlling the monitored function, the subject will indirectly control the pathological situation related to it. Therefore, the proposed system consists of a VR environment in which the subject is placed in front of an avatar who reproduces his features, like a mirror. The identification of the subject in the avatar is reinforced by the use of wireless controllers or (alternatively) by the direct recognition of the hands through the cameras integrated into the headset: by moving the hands the subject will control those of the avatar that represents him. The overall appearance of the avatar will be made dynamic: while maintaining a similarity with the subject, the avatar will be able to change its status from underweight to overweight continuously.

**Figure 4:**
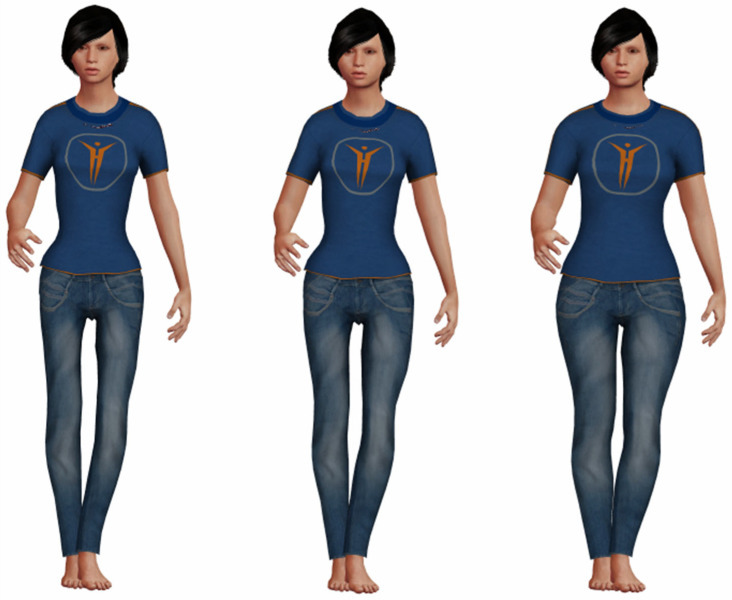
Example of avatar of progressively increasing BMI in the proposed VR application.

The subject will wear a simple galvanic skin response (GSR) sensor, capable of detecting skin conductivity. The implementation of a new instrument to treat AN patient in relation to BI disturbances implies a specific training protocol to control GSR using the feedback provided by the VR application. This application can be used to support the traditional clinical program specifically for AN treatment. As known in the literature [66,67], positive variations in conductivity are indicative of stressful situations, while negative ones show a tendency towards states of relaxation. The system will link the increase in conductivity to a reduction in the BMI of the avatar, while a tendency to relax will be linked to its increase in BMI (Figure 4). A relationship will then be introduced between an emotional state of well-being and the vision of the representation of one’s body in its optimal state, linking the state of tension to its underweight representation instead. The goal, which can be reached through a series of training sessions, is to maintain this perceptual relationship even in daily life.

## 6. Conclusions

As Brown and colleagues outlined in a 2020 paper [68], new treatment options for EDs are needed to enhance treatment outcomes and reduce the rates of dropouts. Integrating VR with existing evidence-based treatments in comprehensive clinical protocol may lead to a more complete understanding of the potential of the integrations of VR into mental health care. Addressing clinical and technological guidelines in a treatment protocol including outcomes data, frequency and number of sessions, and parameters regarding the suitability, safety and acceptability, including cybersickness [69,70] is a fundamental issue. Future research should take advantage of the unique methods of data collection and assessment available within VR, including the collection of biological measurements. Moreover, implementing VR in clinical settings may also foster increased patient participation, also through their involvement in designing VR-based tools; a patient’s experience of success within VR may increase the self-efficacy perception, enhancing confidence to real-world experiences [71]. VR is increasingly being applied in the evaluation and management of patients with AN. This technology allows patients to be immersed in virtual environments that are adapted to their psychological state and can be particularly palatable and interesting for adolescent participants when presented as a game.

The idea that represented the prerequisite for the present research was to start conceptualizing a methodology to implement and test the proposed VR instrument and its validity on adolescents with AN-R in charge at two clinical centres affiliated with the public national health system: The Stella Maris Scientific Institute (IRCCS Stella Maris Foundation, Pisa, Italy) and the “Orti di A.D.A.”, Gardens of A.D.A., a therapeutic-rehabilitation residential structure for minors diagnosed with AN (Pisa, Italy). Furthermore, more specific virtual reality devices could be developed considering the features related to body perception in adolescence. For example, it would be important to focus the attention on body areas particularly affected by the misperception (abdomen, thighs, wrists, cheeks); or it would be important to create devices that consider the changes in the body areas related to puberty (breast growth, enlargement of the hips). A significant development in the future should also be devices that reflect the male body misperception, more and more frequent in the new clinical populations.

The standard multidisciplinary approach in the treatment of AN (psychiatric, psychological, nutritional) could therefore be profitably integrated with these techniques using measures to evaluate their impact on the specific target (body image) and on the overall clinical evolution areas of the young patient. Based on what emerged from the analysis, the possibility of designing a VR system seems important, not only as a research tool but also and above all as a potential complement to treatments for the disorder. Most specialized treatment centers are unable to purchase several complete VR stations, including headsets, controllers, and computers with suitable sound cards. Often these systems need technicians to assist the operators specialized in the treatment. However, in recent years, all-in-one headsets have been available on the market, with more than adequate power to support VR environments such as those described in the articles examined. These devices, based on the Android platform, are straightforward to use and do not require technical skills for their correct operation, connecting third party accessories etc. Oculus Quest 2, for example, comes with two controllers with which it would be immediate to build, for example, third body illusion experiences. This device can autonomously recognize the user’s hands, projecting them into the virtual world. The appearance of the hands is customizable, and it would be very easy to create the illusion of having the typical hands of a fatter or thinner individual so that you can experiment with interaction patterns. Working in an interdisciplinary team that includes clinical staff and experts in advanced technologies, a treatment-oriented experimental proposal may be enriched by this type of device, especially when the fake body illusion paradigm/method is used.

## Figures and Tables

**Table 1:** Relevant literature in body image perception, anorexia nervosa and virtual reality therapeutic applications.

First Author, Year, Institution, Title, Journal	Population	Method	VR, Hardware and Software (HW/SW), Tools and View	Objective	Results	Quality Score
Irvine–2020 Department of Psychology, Faculty of Health and Life Sciences, Northumbria University, Newcastle upon Tyne, UK Using Immersive Virtual Reality to Modify Body Image Body Image Journal	60 female volunteers with high body image concerns.	The participants, divided into two groups, completed a 4-day program, in which they classified a series of 3D models as thin or fat; stimuli were presented to one group briefly, while no time limits were imposed on the other group. Both intervention groups received inflationary feedback to shift their model ratings towards higher BMIs.	VR HW tool: Oculus Rift. Additional HW, notes on SW: Unreal Engine. View: TPV.	Intervening on the perceptual boundary of the classification of a fat body versus a lean body with the use of a VR application.	Both intervention groups experienced statistically significant reductions in their concerns about their body shape, weight, and eating habits. These reductions had clinically significant effects in the group with longer stimulus presentation times.	9/9
Neyret–2020 Event Lab, Department of Clinical Psychology and Psychobiology, University of Barcelona, Barcelona, Spain. Which Body Would You Like to Have? The Impact of Embodied Perspective on Body Perception and Body Evaluation in Immersive Virtual Reality Frontiers in Robotics and AI Journal	Female participants	Participants’ bodies are scanned and generated as avatars. For each body, two more are generated by increasing or decreasing their size. After that, the participants must choose which is their body among the three proposed, both an FPV and TPV mode.	VR HW tool: nVision SX11. Additional HW, notes on SW: Optitrack Motion capture System (a Velcro suit with 28 reflective markers tracked by 12 infrared cameras); tactile feedback through four vibrators controlled by an Arduino board. View: FPV & TPV.	Measure the internal conscious representation of one’s body aspect, comparing it with one’s ideal body aspect and with the real aspect.	Female participants rated their real body as more attractive when they viewed it from a third-person perspective. Their level of dissatisfaction with the body decreased after the experimental procedure. It is hypothesized that the third-person perspective allowed them to perceive their real body shape without applying the previous negative beliefs associated with the self.	9/9
Provenzano–2020 Department of Psychology, “Sapienza” University of Rome, 00185 Rome, Italy and IRCCS, Santa Lucia Foundation, 00142 Rome, Italy Characterizing Body Image Distortion and Bodily Self-Plasticity in Anorexia Nervosa via Visuo-Tactile Stimulation in Virtual Reality Journal of Clinical Medicine	20 anorexics, 20 healthy controls.	For each participant, customized avatars were created, one reproducing their real body size, and two others with increased and decreased weight. Body overestimation and dissatisfaction were measured by asking participants to choose the avatar, presented in FPV, they felt most closely resembled their own body. The activity is completed by a synchronous and asynchronous stimulation with the three versions of the avatar.	VR HW tool: Oculus Rift Developers Kit Dk1. Additional HW, notes on SW: Makehuman (for character creation), Photoshop 7 for skin and dresses, 3dsMax for generation of an avatar with different weights starting from the ones created with Makehuman. View: FPV.	Combining virtual reality and multisensory body illusion to characterize and reduce body overestimation and body dissatisfaction.	Upper body dissatisfaction was found in subjects with AN. The embodiment was stronger with synchronous stimulation in both groups but still did not reduce BI disorder in participants with AN. Subjects with AN reported more negative emotions after embodying the fatter avatar. The cognitive-emotional component, rather than perceptive, of the BID, was severely altered in AN.	9/9
Porras-Garcia–2020 Department of Clinical Psychology and Psychobiology, University of Barcelona, Barcelona, Spain Virtual Reality Body Exposure Therapy for Anorexia Nervosa. A Case Report with Follow-Up Results Frontiers in Psychology	A case study with a patient diagnosed with AN.	The activity included 5 VR therapy sessions. The sessions included exposure in TPV of the patient to a virtual representation of his own body, with the body mass index of the avatar progressively increasing. The virtual environment consists of a room with a large mirror capable of reflecting the image of the body. During the sessions, the participants received synchronous visual-motor and visual-tactile stimulation. Fear of gaining weight (FGW), body anxiety, thinness drive, body image disturbances, body mass index, and attention bias were assessed before and after treatment, as well as 5 months after.	VR HW tool: HTC-VIVE. Additional HW, notes on SW: VR HMD FOVE Eye Tracking to detect and register eye movements, Unity 3D and Blender for avatar creation. View: FPV.	This study aimed to provide preliminary evidence of the usefulness of VR body exposure therapy.	After the treatment, there was a reduction in typical AN symptoms such as FGW, thinness drive, body-related anxiety, and dissatisfaction. There was a noticeable change in body-related dysfunctional attention bias. Thanks to the intervention, the values of the body mass index have increased and reached healthy levels. Most of all these improvements were maintained after 5 months.	8/8
Porras-Garcia–2020 Department of Clinical Psychology and Psychobiology, University of Barcelona, Barcelona, Spain Virtual Reality Body Exposure Therapy for Anorexia Nervosa. A Single Case Study In: Stephanidis C., Antona M. (eds) HCI International 2020–Posters. HCII 2020. Communications in Computer and Information Science	A case study on a 14-year-old female adolescent with AN.	A virtual representation of the patient’s body was reconstructed, whose weight was progressively increased in 5 sessions. The avatar was displayed to the patient in a simple VR setting. FGW, Body Anxiety, and Full Body Illusion (FBI) were assessed at the start of each session. Fear of gaining weight (FGW), body anxiety, thinness drive, body image disorder (BID), and body mass index (BMI) were assessed before, after treatment, and 3 months after follow-up. -up of 3 months.	VR HW tool: HTC-VIVE. Additional HW, notes on SW: VR HMD FOVE Eye Tracking to detect and register eye movements, Unity 3D and Blender for avatar creation. View: FPV.	Traditional exposure-based therapies have significant limitations (for example, the negative initial reaction in patients or increased risk of abandonment). The use of VR-based exposure techniques can overcome these limitations.	After the treatment they decreased in FGW, push to thinness, body-related anxiety, and BID. BMI slightly increased throughout the surgery. FBI levels also increased progressively with each exposure session. However, these changes were not fully maintained at follow-up.	8/8
Fisher–2020 Child and Adolescent Psychopathology Unit, Salvator University Hospital, Public Assistance-Marseille Hospitals, Aix-Marseille University, 249 Boulevard Sainte-Marguerite, 13009 Marseille, France Comparison of body image evaluation by virtual reality and paper-based figure rating scales in adolescents with anorexia nervosa: retrospective study Eating and Weight Disorders–Studies on Anorexia, Bulimia and Obesity Journal	31 female adolescents with AN.	Ten 3D avatars are created, with different builds, arranged in a circle. The participant observes the scene in VR while the observation time of each of the avatars is measured. Next, the participant has to choose the avatar that best matches their current image and the avatar that matches their desired shape. Paired data of perceived and desired body shapes were compared as well as body perception index (BPI) (*p* = 0.2) and body dissatisfaction (*p* = 0.6). The correlation of the data found with validated psychometric questionnaires was measured.	VR HW tool: Oculus Rift. Additional HW, notes on SW: C2CARE PSY (application realized with Unity). View: TPV.	The objective of the study is to demonstrate that VR systems with standardized avatars would improve the perception of the body image and therefore the evaluation of the body image by subjects with AN, compared to the evaluation scales of the figures on paper (FRS).	Participants with AN overestimated their body size regardless of the assessment tool used. BPI and body dissatisfaction did not differ significantly between FRS and VR. The results of the BID evaluation by VR standardized 3D avatars are comparable to those obtained by paper FRS.	7/8
Hudson–2020 Department of Health, Kinesiology and Sport, University of South Alabama, USA The Development of a BMI-Guided Shape Morphing Technique and the Effects of an Individualized Figure Rating Scale on Self-Perception of Body Size European Journal of Investigation in Health Psychology and Education	Young adult women	A corresponding avatar was created for each subject, along with 3 others with slightly different apparent weights. The avatars were then shown, in a 2D and 3D version, in a VR setting in TPV mode.	VR HW tool: Oculus Rift. Additional HW, notes on SW: Two Microsoft Kinect v2 for body scanning. View: TPV.	The study aimed to assess the effects of an Individualized Figure Rating Scale on Self-Perception of Body Size	Assessments of body perception using generalized line drawings (2D) were often superior to responses using individualized visualization methods. The realism of representation, therefore, does not seem to be of much importance, except in helping to identify. Using scales based on custom textures and limb sizes seems beneficial, but immersive VR presentation may not be essential.	7/9
Mölbert–2019 Department of Psychosomatic Medicine and Psychotherapy, Medical University Hospital Tübingen, Germany. Assessing Body Image in Anorexia Nervosa Using Biometric Self-Avatars in Virtual Reality: Attitudinal Components Rather than Visual Body Size Estimation Are Distorted Psychological Medicine	24 women with AN and *n* = 24 controls.	Realistic avatars were created for each participant based on a 3D scan. The apparent weight of the avatars was then varied within a range of ±20%. The avatars were then presented to the subjects in a mirror-like scenario, in virtual reality.	VR HW tool: Not specified. Additional HW, notes on SW: 3D scan for avatar generation. View: FPV.	The study, using AR technologies, aimed to separate the perceptual and attitudinal components of the BID into AN.	Women with AN underestimated their weight more than controls. The target mean bodyweight of the controls had normal weight while the target mean weight of the women with AN corresponded to the extreme AN (DSM-5). Desired body weight was found to be associated with eating disorder symptoms.	9/9
Rubo and Gamer–2019 Marcusstr. 9–11, D-97080 Wuerzburg, Germany Visuo-Tactile Congruency Influences the Body Schema during Full Body Ownership Illusion Consciousness and Cognition	24 women with AN diagnosed according to DSM-5 and *n* = 24 age and gender-matched normal weight control.	A corresponding avatar has been created for each subject. The apparent weight of the avatar has therefore been slightly increased. After the calibration phase, the participant observes himself through a VR mirror and has to perform simple tasks such as touching his hips and stomach and walking around a table.	VR HW tool: HTC Vive. Additional HW, notes on SW: TPCAST Wireless Adapter instead of a standard cable to allow participants to move more freely. Unity 3D. Unity Humanoid AdjustProportion and Unity Vertex Displacements (two in-house software to adjust human body proportions and to apply distortion to the Euclidean space of the virtual scene. View: FPV&TPV.	The aim was to assess if visuotactile congruency influences the body schema during VR-based full body ownership illusion.	Participants who took possession of a more corpulent virtual body with visual-tactile congruence set at the normal level increased safety distances to the laboratory walls compared to participants who experienced the same illusion with limited visual-tactile congruence.	9/9
Porras-Garcia–2019 Department of Clinical Psychology and Psychobiology, University of Barcelona, Barcelona, Spain The Influence of Gender and Body Dissatisfaction on body-related Attentional Bias: An Eye-Tracking and Virtual Reality Study International Journal of Eating Disorders	Forty-five women (23 with high BD and 22 with low BD) and 40 men (20 with high BD and 20 with low BD).	Participants were represented in three virtual avatars, the first based on the participant’s actual measurements, the second larger than the participant, and the third with an apparent weight similar to the first avatar. The number of fixations and full fixation time on weight-related areas of interest (W-AOI) and no weight-related areas of interest (NW-AOI) were recorded for the three assessment times/avatars.	VR HW tool: HTC-VIVE. Additional HW, notes on SW: VR HMD FOVE Eye Tracking to detect and register eye movements, Unity 3D and Blender for avatar creation. View: TPV	The aim was to assess gender differences in attentional bias (AB) towards weight-related and non-weight-related body parts, using virtual reality and eye-tracking techniques.	The results showed an interaction between sex, total time, and several fixations. BD levels did not significantly affect the results. Overall, women paid more attention to weight-related areas of interest (W-AOI) than men. Additionally, preliminary evidence was found for increased attention to muscle-related areas of the body in male subjects.	9/9
Serino–2019 IRCCS Istituto Auxologico Italiano-Catholic University of the Sacred Heart From Avatars to Body Swapping: The Use of Virtual Reality for Assessing and Treating Body-Size Distortion in Individuals with Anorexia Journal of Clinical Psychology	A patient with a diagnosis of AN (DSM-5) underwent intensive multidisciplinary outpatient treatment.	The protocol was composed of three sessions on the body-exchange paradigm in VR, obtained through synchronous and asynchronous visual-tactile stimulations.	VR HW tool: Oculus Rift SDK2. Additional HW, notes on SW: Makehuman to generate the avatar. Unity 3D to generate the virtual scene. View: FPV.	This case study aimed to use body illusion techniques in a VR protocol within a multidisciplinary treatment of AN.	The Full Body Illusion (FBI) in VR environment has proven effective in monitoring changes in multisensory body integration. The study also demonstrates how the FBI can help foster these changes.	8/8
Fonseca-Baeza–2018 Universitat de València, Valencia, España An Intervention Protocol Proposal to Modify the Body Image Disturbance Using Virtual Reality Calidad de Vida y Salud	A community sample of young women (18–35 years old), having a BMI between 18.5 and 24.99.	A standard female virtual body (with hidden hair and face) was developed for all participants, shown in VR in FPV and TPV modes. The avatar is initially proposed with an apparent BMI similar to that of the subject. The participant is then asked to change the avatar until the avatar’s abdomen matches her real abdomen.	VR HW tool: Oculus Rift. Additional HW, notes on SW: TANITA to measure the weight and analyze the body composition. Makehuman was used to generate the avatar. View: FPV and TPV.	The study aimed to present a study protocol of virtual reality (VR) multisensory paradigm to evaluate and treat BID.	This protocol appears to be an effective tool for developing a more realistic body representation of oneself.	8/9
Corno–2018 Université du Québec en Outaouais Gatineau, Québec, Canada Assessing the Relationship Between Attitudinal and Perceptual Component of Body Image Disturbance Using Virtual Reality Cyberpsychology Behavior and Social Networking	A sample of 27 community women.	Starting with a body with standard BMI, (presented in VR in both FPV and TPV modes) subjects must indicate how to modify it to make it look like their body. The women were able to choose from a wide range of three-dimensional bodies with a body mass index between 12.5 and 42.5 kg/m^2^. Standard indices such as body dissatisfaction, body discomfort, and avoidance of body image were assessed through questionnaires.	VR HW tool: Oculus Rift SDK2. Additional HW, notes on SW: Makehuman for avatar generation. Unity 3D for scene generation. Kinect V2 to detect participant movement. View: FPVandTPV.	This study aimed to explore the attitudinal and perceptual components of the BID using VR technologies.	The study showed that the attitudinal components predicted the BID only in the TPV mode. Overestimation was predicted by body image avoidance, while underestimation was predicted by body discomfort. Furthermore, a common predictor of underestimation and overestimation was body dissatisfaction.	8/9
Buche and Le Bigot–2018 LAB-STICC, ENIB and LAB-STICC, UBO REVAM: A Virtual Reality Application for Inducing Body Size Perception Modifications In Proceedings-2018 International Conference on Cyberworlds	16 female participants.	The REVAM application proposes to couple a tactile stimulation while viewing a TPV avatar. In addition, the application offers the possibility to choose between avatars of different sizes and to morph between them. The experiment consists in estimating the affordance, in a basic situation and in another 4 in which visual-tactile stimulation is present.	VR HW tool: Oculus Rift Dk2. Additional HW, notes on SW: Kinect, Razer Hydra. View: TPV.	The study aims to propose a 3D virtual environment to induce the illusion of body ownership (whole body illusion).	It was noted that the average width of a door represented in VR, through which participants estimate they can pass with the avatar representing them, was significantly reduced when morphing was present. Simultaneous tactile stimulation does not appear to affect this result. The study speculates that exposing people to a smaller virtual body in this way could be an effective way to change the perception of body size, at least temporarily.	8/9
Serino–2017 IRCCS Istituto Auxologico Italiano-Catholic University of the Sacred Heart Two-phases innovative treatment for anorexia nervosa: The potential of virtual reality body-swap Annual Review of Cyber Therapy and Telemedicine	A group of23 female participants suffering from Anorexia Nervosa.	Participants observed in VR, in FPV mode, a virtual body with a lean belly representing their physical body in two experimental conditions: synchronous and asynchronous visual-tactile stimulation.	VR HW tool: Oculus Rift DK2. Additional HW, notes on SW: Razer Hydra Portal 2. View: FPV.	The goal is to investigate whether a treatment based on VR-based full-body illusion can induce variations in the representation of the body.	The study showed that the treatment can reduce the decrease in body distortions in the abdomen. This VR-based approach, if further developed, may be useful for anorexic patients to specifically improve body representation disorders.	8/9
Ferrer-Garcia–2017 Department of Clinical Psychology and Psychobiology, Universitat de Barcelona, Barcelona, Spain Does Owning a ‘Fatter’ Virtual Body Increase Body Anxiety in College Students? Annual Review of CyberTherapy and Telemedicine	23 college students (5 male).	The subjects were exposed to an immersive VR environment in FPV and TPV modes. For each participant, an avatar with compatible apparent body measurements was reconstructed, plus two other avatars, respectively, one heavier by 20% and one lighter by 20%. Before exposure, BMI, drive to thinness (EDI 3-DT) and body dissatisfaction (EDI3-BD) were assessed. Body anxiety (PASTAS), fear of gaining weight, and delusion property (VAS 0 to 100) were assessed after exposure to each avatar. The illusion of ownership of a virtual body was induced through visual-motor synchronization.	VR HW tool: HTC-Vive. View: FPV and TPV.	Evaluate the ability of virtual reality (VR) based software to produce body anxiety responses in a non-clinical sample.	The study showed that the virtual body illusion with measurements larger than the subject’s causes body anxiety and fear of gaining weight in subjects with higher body dissatisfaction. BMI did not affect the results.	8/9
Cipolletta–2017 Department of General Psychology, University of Padua Intrapersonal, interpersonal, and physical space in anorexia nervosa: a virtual reality and repertory grid investigation Psychiatry Research	A sample of 12 AN patients and 12 HCs (controls) participated in the study.	The Eating Disorder Inventory (EDI), a procedure based on virtual reality, traditional measures of spatial skills, and repertoire grids were used in the protocol.	VR HW tool: -Additional HW, notes on SW: Logitech 940-000114 F510. NeuroVirtual 3D. View: FPV and TPV	This study, based on VR technologies, aimed to verify the differences between the spatial perception of patients with AN from healthy controls, and are these differences related to the severity of the anorexic symptoms.	The study showed that AN subjects showed significant impairments in spatial abilities, more unidimensional construing, and more extreme construing of the present self and the self as seen by others when compared to controls.	9/9
Keizer–2016 Experimental Psychology/Helmholtz Institute, Utrecht University, Utrecht, The Netherlands A Virtual Reality Full Body Illusion Improves Body Image Disturbance in Anorexia Nervosa Plos One	AN patient (*n* = 30) and HC group (*n* = 29).	Participants were asked to estimate their body size (shoulders, abdomen, hips) before Full Body Illusion (FBI) was induced, immediately after induction, and approximately 2 h and 45 min of follow-up. Full Body Illusion in VR was induced through synchronous visual-tactile stimulation.	VR HW tool: Oculus Rift DK2. Additional HW, notes on SW: Razer Hydra Portal 2. Unity 3D, MakeHuman. View: FPV	The study aimed to investigate whether a virtual reality-based full-body illusion affects the body size estimate of various body parts.	AN patient decreased the overestimation of their shoulders, abdomen, and hips directly after the FBI was induced. The effect was noted also in controls, although with a different pattern.	9/9
Malighetti–2016 Department of General Psychology, University of Padua Inside and outside the self. Virtual reality and repertory grids in the spatial analysis of anorexic patients’ meanings Annual Review of Cyber Therapy and Telemedicine	A sample composed of 12 AN patients and 12 healthy controls.	In a first task, the participants were asked to find a hidden object and memorize its position in a virtual city. After they have found the object, the second task participants were invited to retrieve the position of the object, which was absent, after entering the virtual city from another starting point.	VR HW tool: Oculus Rift DK2. Additional HW, notes on SW: Razer Hydra Portal 2. View: FPV.	The general objective of this study was to investigate, through a system based on VR technologies, the presence of deficits in the elaboration of the egocentric and allocentric frame of reference in AN patients.	The AN patient showed deficits in the spatial ability to retrieve and update a long-term memorized representation. It is hypothesized that the deficit in processing spatial information could be related to a distorted body representation.	8/9
Serino–2015 Applied Technology for Neuro-Psychology Lab, IRCCS Istituto Auxologico Italiano, Via Magnasco 2, 20149 Milan, Italy Out of Body, out of Space: Impaired Reference Frame Processing in Eating Disorders Psychiatry Research	Healthy controls/ED (eating disorder) patients	In a preliminary phase, the Corsi Block Test- Span and Supraspan (Corsi, 1972), and the Judgment of Line Orientation (Benton et al., 1978) were used to assess visuospatial abilities. Participants were then invited to retrieve the position of a hidden object they had discovered in the virtual city and memorized it on a real map (an aerial view of the virtual city) with a pen (allocentric view). In a second phase, they have to indicate the position of that object (which was absent) entering the virtual city from another starting point (egocentric view).	VR HW tool: Oculus Rift DK2. Additional HW, notes on SW: Razer Hydra Portal 2. View: FPV.	The aim was to investigate the relationship between ED and spatial abilities.	Employing a well-validated virtual reality-based procedure related to healthy controls, patients with eating disorders showed deficits in the ability to reference and update a long-term stored allocentric representation (TPV) with egocentric input (FPV).	8/9
Conxa Perpiñá and Botella–2013 San Vicente Mártir Catholic University of Valencia Effectiveness of Cognitive Behavioral Therapy Supported by Virtual Reality in the Treatment of Body Image in Eating Disorders: One Year Follow-Up Psychiatry Research	34 participants diagnosed with eating disorders.	The treatment consisted of 15 sessions divided into 3 stages. The virtual environment consisted of 5 different areas. Area 1: Virtual scale and kitchen. Area 2: Photograph area, Here the patient’s tendency to compare themselves with other bodies is addressed. Area 3: Mirror room. The patient has to manipulate a 3D avatar until it matches a 2D image representing his/her real body. Area 4: a sort of adjustable door, the user has to set it so that he/her can pass through it. Area 5: several versions of one’s body are here presented. The is invited to compare them.	VR HW tool: Virtual Research V6. View: FPV/TPV.	Testing effectiveness of cognitive behavioral therapy supported by virtual reality.	Patients who received the described treatment improved their body image. The improvement was also maintained in the follow-up.	8/9
Maldonado–2010 Department of Personality, Assessment and Psychological Treatments, University of Barcelona, Barcelona, Spain Body Image in Eating Disorders: The Influence of Exposure to Virtual-Reality Environments Cyberpsychology Behavior And Social Networking	85 ED patients and 108 non-ED students.	VEs to simulate real-life situations that were emotionally significant for ED patients, to produce or enhance body-image distortion and body-image dissatisfaction. The subjects have been exposed to high-calorie food, as well as situations in which their body is on display or in which they come into contact with other people.	VR HW tool: HTC-Vive. View: FPV and TPV.	The study aimed to evaluate the effect of virtual-reality exposure to situations that are emotionally significant for patients with eating disorders (ED) on the stability of body-image distortion and body-image dissatisfaction.	The study shows the presence of a distorted body image in patients compared to controls and highlights the fact that, in the patient group, these body images vary according to the emotional and social situation.	8/9
Riva–1999 Applied Technology for Neuro-Psychology Lab., Istituto Auxologico Italiano, Verbania, Italy. Virtual reality-based experiential cognitive treatment of anorexia nervosa Journal of Behavior Therapy and Experimental Psychiatry	Case study on a 22-year old female diagnosed with AN.	The study used experiential cognitive therapy (ECT), with the use of VR technologies. The treatment consisted of 5 sessions with the VEBIM virtual reality system. The first session was used to assess any stimuli that could elicit abnormal eating behaviour. The next four sessions were used to assess and modify: the symptoms of anxiety-related to food exposure and the body experience of the subject. All the sessions were guided by a therapist.	VR HW tool: Thunder 400/C virtual reality system by Virtual Engineering of Milano-Italy. View: FPV	The work describes the treatment, with VR technologies, of a 22-year-old university student diagnosed with Anorexia Nervosa	The patient had a high degree of motivation to change. The subject increased his body awareness coupled with a reduction in his level of body dissatisfaction.	8/8
Riva–1998 Applied Technology for Neuro-Psychology Lab., Istituto Auxologico Italiano, Verbania, Italy. Experiential Cognitive Therapy in Anorexia Nervosa Eating and Weight Disorders	Case study on a 22-year old female diagnosed with AN.	The study used experiential cognitive therapy (ECT), with the use of VR technologies (see 24).	VR HW tool: Thunder 400/C virtual reality system by Virtual Engineering of Milano-Italy. Additional HW, notes on SW: two-button joystick-type motion-input device. View: FPV	The article describes the characteristics of experiential cognitive therapy (ECT): a short-term, integrated and patient-oriented approach that focuses on individual discovery.	The patient had a high degree of motivation to change. The subject increased his body awareness coupled with a reduction in his level of body dissatisfaction.	8/8
Riva–1997 Applied Technology for Neuro-Psychology Lab., Istituto Auxologico Italiano, Verbania, Italy. The Virtual Environment for Body-Image Modification (VEBIM): Development and Preliminary EvaluationPresence-Teleoperators And Virtual Environments	72 normal subjects.	The treatment uses a VR environment built with a VEBIM system. It consisted of a visit to 6 zones, guided by a therapist. Zone 1: a virtual balance. Zone: interaction with food. To move to zone 3 subjects had to weigh again. Zone 3: a corridor with male and female models. Zone 4: a room with the subject’s digitized picture. Zone 5: a room with four doors of different dimensions. The subject can only move into the by choosing the door corresponding exactly to his width and height. Zone 6: a room with the image of the subject’s real body, plus an image that can be modified to use to last zone create its ideal body.	VR HW tool: Thunder 400/C virtual reality system by Virtual Engineering of Milano-Italy. Additional HW, notes on SW: two-button joystick-type motion-input device. View: FPV	The purpose of the article is to describe the VEBIM (Virtual Environment for Body-Image Modification) theoretical approach and its characteristics.	The results report significantly lower values for FRS (Figure Rating Scale, Thompson, and Altabe, 1991) and CDRS (The Contour Drawing Rating Scale, Thompson, and Gray, 1995) after the experience in the virtual environment.	8/9

NOTE: Quality score was based on the Newcastle-Ottawa Scale (NOS) and on the scale published by Murad et al., 2017; FPV: First Person View; TPV: Third person View; MHD: Head Mounted Display; FBI: Full Body Illusion; BID: Body Image Disorder [38].
